# A Method for the Colorimetric Quantification of Sodium Lauryl Sulphate in Tablets: A Proof of Concept

**DOI:** 10.3390/pharmaceutics16081100

**Published:** 2024-08-21

**Authors:** Artūrs Paulausks, Austris Mazurs, Valentyn Mohylyuk

**Affiliations:** Leading Research Group, Faculty of Pharmacy, Rīga Stradiņš University, LV-1007 Riga, Latvia

**Keywords:** sodium lauryl sulphate, spectrometric determination, tablet dissolution, quantification, matrix effects, deformulation

## Abstract

The deformulation stage of original drug products, which includes the quantification of critical excipients, is crucial for the successful development of generic drug products of solid dosage form. Sodium lauryl sulphate (SLS) belongs to the group of critical excipients due to its influence on the bioavailability of drugs, such as metformin. The purpose of this work is to carry out a feasibility study in order to develop a simple, economical, and robust analytical method for the quantification of SLS in metformin-containing tablets after their dissolution in water. Firstly, SLS is extracted with chloroform in acidic conditions, followed by the addition of methylene blue (MB) in order to form a SLS-MB ion pair, which is then measured photometrically at a wavelength of 651 nm. Additionally, interference from matrix components (excipients and APIs) was assessed, and it was found that metformin also forms a blue complex; therefore, this specific extraction method was developed. Other matrix components did not interfere with SLS determination. This method shows a well-estimated precision of 3.3% and accuracy of 5%, a calibration linearity of R^2^ = 0.99990, and a working range of 0.38 µg/mL to 10 µg/mL of SLS in water. The midpoint of the calibration graph corresponds to the concentration of SLS obtained by dissolving a single tablet in 1 L of water. This method seems appropriate for total SLS determination in tablets and can be applicable for deformulation.

## 1. Introduction

The industry of generic drug manufacturing plays an essential role in the national healthcare systems. It allows for an increase in the availability of medication for a wider population to decreases the financial burden and simultaneously retains the same level of safety and efficacy as an original drug product. To claim the same indications as an original drug product, the generic (test) product should prove and justify the similarity to the original one [1]. Based on the drug solubility and permeability, as well as drug release kinetics from the reference product, the justification of test-to-reference product similarity can include or exclude very expensive and resourceful bioequivalence studies involving healthy volunteers [1].

Sodium lauryl sulphate (SLS), also known as sodium dodecyl sulphate, can be found in different forms but mostly in oral dosage forms [2] (Supplementary Table S1). SLS is used in tablet formulations in the concentration range of 0.1–1.5 wt.% [3] to improve the tablet wettability and apparent solubility of drug substances. Additionally, it can act as a lubricant to reduce friction and adhesion within the die during tablet pressing [4]. According to the U.S. Food and Drug Administration, SLS was claimed as a lubricant twenty times in Abbreviated New Drug Application [5]. Importantly, SLS can change oral bioavailability by increasing the apparent solubility of the drug and influencing transepithelial transport [6,7]. SLS, at a concentration range of 0.025–1.0%, showed an ex vivo dose-related permeability increase through the canine oral mucosa for twelve organic compounds [8]. The underlying mechanism of the SLS-mediated enhanced permeability was explained by the reversible opening of tight junctions [7]. SLS can increase the paracellular transport of metformin via human colorectal adenocarcinoma (Caco-2) cells which mimic the intestinal epithelium: the initial permeability of metformin (1.36 × 10^−5^ ± 1.25 × 10^−6^ cm/s) was increased 1.9-fold at an SLS concentration of 0.012% (*w*/*v*) [9].

To achieve test-to-reference product composition and property similarity, the development of the generic product often includes a deformulation stage of the original product. This stage includes the quantification of critical excipients such as SLS. This allows for shortening of the formulation development based on the in vitro property’s similarity (such as disintegration time and dissolution profile). Additionally, deformulation increases the success rate of bioequivalence studies because the in vitro property’s similarity cannot guarantee the in vivo similarity.

Very often, tablets are manufactured with a coating to provide visual differentiation, to improve stability, or to mask the taste. Coatings contain several excipients, such as sugars, polymers, plasticizers, surfactants, antifoaming agents, organic dyes, and inorganic pigments. If a scientist is interested in the composition of a tablet core, it is highly recommended that they remove the coating from the tablet’s surface. If the component of interest is distributed evenly in the core, a part of the core (without coating) with a known mass can be used. Otherwise, if the distribution is unknown, careful removal of the coating and the use of the whole core is recommended. The removal of the coating will decrease the chemical complexity of the tested object, uncertainty, and the probability of undesirable chemical interactions upon quantification.

In order to determine the SLS content in a tablet, several methods have been proposed. These include gas chromatography (GC) methods which require a derivatization step to convert SLS to lauryl alcohol, which then can be used to determine directly [10,11,12] or derivatize further with silylating agents [13]. These methods involve tedious sample preparations, where the sample is heated to 80 °C within acidic conditions for up to several hours to achieve SLS conversion. Also, not all laboratories are equipped with the required equipment for sample preparation or GC itself. Methods using high-performance liquid chromatography (HPLC) with ultra-violet (UV) spectroscopy detection were not found, which is likely due to the lack of chromophores within the SLS molecules. While it is possible to use liquid chromatography-mass spectrometry (LC-MS) to determine SLS [14], it being more selective would require expensive equipment. Alternatively, chromogenic [15,16], fluorescence [17], and some electrochemical methods are also proposed [18].

SLS is a critical excipient that can influence the bioavailability of drugs (such as metformin). Thus, precisely determining its concentration is tremendously important in generic product development. This study aims to undertake a feasibility study in order to develop a simple, economical, and robust analytical method for the quantification of SLS in tablets containing metformin.

## 2. Experimental Part

### 2.1. Chemicals and Materials

Chloroform was obtained from Fisher Chemical, ≥99.8%, stabilised with amylene. Purified water was obtained from a StakPure Omnia Tap 6 water purifier (Stakpure, Berlin, Germany), with a conductivity of 18.2 MΩ × m. Sodium sulphate (ACS reagent, ≥99.0%, anhydrous, powder) and sulfuric acid (ACS reagent, 95.0–98.0%) were obtained from Sigma-Aldrich (Merck KGaA, Darmstadt, Germany). Methylene blue (98.0%) was obtained from Tokyo Chemical Industry Co., Ltd. (Tokyo, Japan). Sodium lauryl sulphate (SLS) was provided by BASF SE (Ludwigshafen am Rhein, Germany). The methylene blue reagent contained 12 mg methylene blue, 2.5 g sodium sulphate anhydrous, and 0.5 mL sulfuric acid in 50 mL of purified water, prepared in our laboratory using the above-mentioned ingredients. In the same laboratory, an acidified sodium sulphate solution was prepared by mixing 25 mL of saturated sodium sulphate solution in purified water and 25 mL of sulfuric acid. JANUMET^®^ tablets (Merck Sharp & Dohme Idea Inc., Haarlem, The Netherlands; Table 1) contained SLS and metformin.

### 2.2. Sample Preparation

Tablets with the coating were weighed, after which the coating was removed using a scalpel and the tablets were weighed once again. After the removal of the coating, each of the tablets was placed into a 1 L beaker with a magnetic stirrer, to which 1 L of purified water was added. The tablets were allowed to dissolve over 24 h under gentle stirring (to allow soluble components to be dissolved; the duration could be optimised). Afterwards, 3 mL of the tablet solution was transferred to a 50 mL polypropylene (PP) centrifuge tube, to which 3 mL of acidified sodium sulphate solution was added. The contents of the centrifuge tube were then vortexed. SLS was extracted by adding 20 mL of chloroform to the solution and mixing via vortex (Intllab VM-370; Shenzhen Jiashi Technology Co., Ltd., Shenzhen, China). The chloroform layer was allowed to settle, and it was transferred to a separate 50 mL PP tube, to which 3 mL of methylene blue reagent was added. The solution was vortexed, and the chloroform layer was allowed to settle.

### 2.3. Calibration Solutions

The concentrated stock solution was prepared by dissolving 50.00 mg of sodium lauryl sulphate in 100.0 mL of water, resulting in a concentration of 500.0 µg/mL (stock solution 1). To prepare the first four calibration levels, the concentrated stock solution was further diluted ten times (1 mL stock solution and 9 mL purified water) to give a diluted solution with a concentration of 50.00 µg/mL (stock solution 2). Calibration solutions were prepared by diluting either stock solution 1 or stock solution 2 with purified water. Eight different concentrations were chosen for calibration. Then, 10 mL of each solution was prepared. The volumes of stock/water used as well as the final concentrations of the calibration solutions are presented in Table 2. The calibration solutions were then prepared and analysed as described in the method.

### 2.4. Spectrophotometry

Absorption was measured against a blank (water extract), which was prepared in the same way as the samples. The measurement was performed at 651 nm in a 5 cm quartz cuvette, using a double-beam spectrophotometer (Shimadzu UV-1900i; Shimadzu, Tokyo, Japan).

### 2.5. Testing of the Method

A calibration graph was constructed in a range of 0.40–10.0 µg/mL. The Limit of Detection (LoD) and Limit of Quantitation (LoQ) were calculated from a linear calibration graph using Equations (1) and (2).

(1) LoD=3.3×Snk

(2) LoQ=10×Snk

where *K* is a slope of the calibration line; *S_n_* is a standard error of the Y-intercept.

Accuracy was approximated by running a sample trial with three formulations (Table 1). Precision was tested by analysing each formulation solution four times. Matrix interferences were tested by comparing calibrations performed with spiked water and those of matrix component solution. In detail, the amount of ingredients from one tablet (Table 1), other than SLS, was added to 1 L of water and stirred. Afterwards, 3 mL of this solution was transferred to a 50 mL PP centrifuge tube and spiked with SLS. In parallel, pure water solutions were also prepared with the same spiking level. All samples were prepared as described in the sample preparation (Section 2.2) and measured against blank water extract.

Matrix effects (MEs), accuracy, and precision were evaluated using Equation (3).

(3) $$ME=\left(1-\frac{K_{w}}{K_{m}}\right)\times 100\%$$

where *K_w_* is a slope of calibration line obtained with spiked water and *K_m_* is a slope of calibration line obtained with spiked matrix solution.

## 3. Results and Discussion

Since the dissolved tablet solutions contain known amounts of matrix components (Table 1), the less selective method can be chosen. Therefore, to avoid contamination of the instrument, complicated derivatization procedures, and high analysis costs, the SLS determination method proposed by Arand et al. was adapted. This method relies on the formation of blue, chloroform-soluble ion pairs of SLS and methylene blue (MB) [15].

Sample preparation was also adopted from Arand et al. [15] for the determination of SLS in the tablet solutions. The tablet was dissolved in large enough volumes of water to avoid SLS micelle formation, since the SLS critical micelle concentration is 5 mg/mL [19]. Furthermore, the order in which the reagents were added to the samples was changed to first selectively extract SLS into chloroform, and then to further turn it into a blue SLS-MB ion pair. The original paper from Arand et al. includes a chloroform drying step with sodium sulphate, but during experiments sticking of some blue residues to the crystals of sodium sulphate was noticed. After adding excess amount of sodium sulphate to the sample, the spectrometric absorbance of SLS-MB decreased by around 20%. This could be due to the SLS-MB ion pair adsorbing on the crystal surface. Because of that, this drying step was excluded and instead chloroform was simply centrifuged or allowed to settle in order to separate it from water. Furthermore, to avoid contamination from residue detergents used to wash regular laboratory glassware, all experiments were conducted using single-use plastic PP centrifuge tubes.

**Figure 1:**
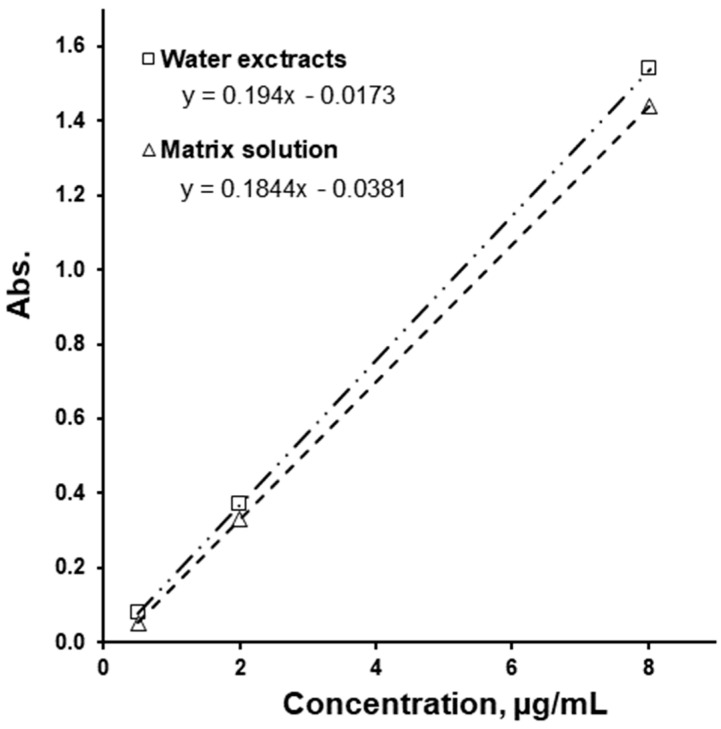
Calibration performed with water compared to calibration performed with matrix solution.

During the method trial, quite severe interference from metformin was noticed, which also formed a blue ion pair with methylene blue. Assuming a 100% extraction efficiency of metformin, it was calculated that the metformin-MB ion pair has a molar extinction coefficient of 4.77 (mol^−1^ × cm^−1^), while for the SLS-MB ion pair this is 47.9 (mol^−1^ × cm^−1^). Since some of the tablets contain up to 1 g of metformin, and this was considered to be a significant interference. Therefore, SLS was first selectively extracted in chloroform by adding sodium sulphate and sulfuric acid solution to the sample. This was performed to obtain a non-polar, protonated SLS form and to increase the ionic strength, which would facilitate SLS extraction into the chloroform. Metformin, being basic (pKa 11.8), forms protonated cations and stays in the water layer. Other matrix components did not cause significant interferences. Finally, testing for matrix interference, a 5% decrease in the slope of the calibration graph was noticed when the matrix components were present (Figure 1).

**Figure 2:**
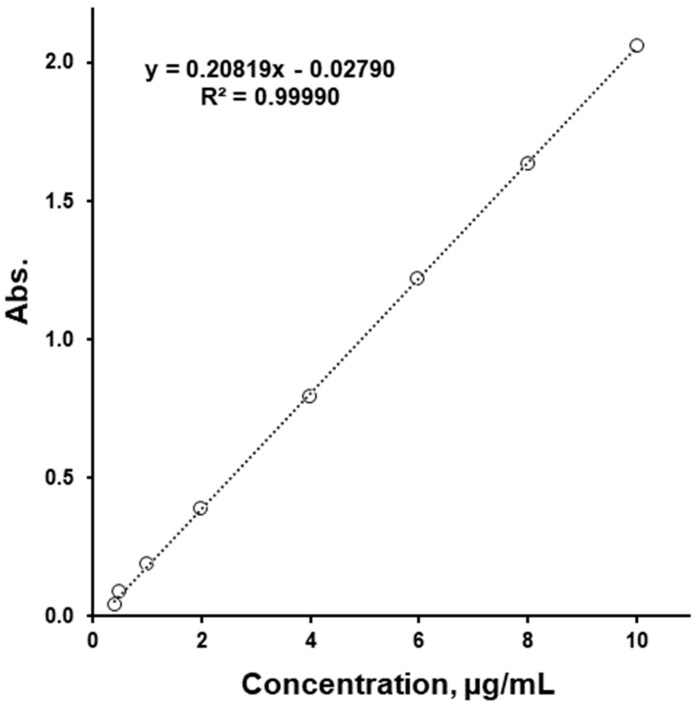
Calibration plot of SLS.

To increase the method sensitivity, a 1 cm cuvette to 5 cm cuvette change was used, which allowed us to decrease the LoQ value by four times. Even though higher calibration samples reached absorption values above two units, the linearity of R^2^ = 0.99990 was excellent (Figure 2). The LoQ and LoD values were calculated using the intercept of this calibration line, giving values of 0.25 and 0.08 µg/mL, respectively. These values are not quite accurate, since using a calibration equation with a negative intercept would give negative absorption values for these concentrations. Therefore, adding 0.13 µg/mL to these values, which is where the calibration line intercepts the X-axis, would give an LoQ of 0.38 µg/mL and an LoD of 0.21 µg/mL. The precision of this method was found to be up to 3.3% RSD, and the accuracy up to 5% RSD. Here, the accuracy by comparing results obtained by this method to a nominal tablet SLS value for three types of tablets (Table 1) was estimated. The results ranged from 1 to 5% accuracy. Using the method developed, the SLS contents in the tablet cores of “50/1000”, “50/850”, and “50/500” were found to be 0.49, 0.47, and 0.49 wt.%, respectively.

## 4. Conclusions

The method for the determination of SLS in tablets containing sitagliptin/metformin was adapted from Arand et al., with some minor but crucial changes in sample preparation. Although this method is not fully validated yet, some of the experiments described here show promising results and serve as a proof of concept. This method can potentially be used for the determination of SLS at the deformulation stage, especially for laboratories with limited equipment. The sensitivity and linearity allow one to determine concentrations of SLS down to a sub-ppm level in water solutions, and with some changes in sample preparation this could possibly go even lower. This methodology for adapting the method described here could allow for the adaption of this method to different samples.

## Figures and Tables

**Table 1:** Expected tablet cores composition.

Ingredients	“50/1000”		“50/850”		“50/500”	
	mg	*w*/*w* %	mg	*w*/*w* %	mg	*w*/*w* %
Sitagliptin phosphate monohydrate	64.3	4.8	64.3	5.6	64.3	9.1
Metformin hydrochloride	1000.0	74.6	850.0	73.9	500.0	70.4
Microcrystaline cellulose	≈137.4	≈10.3	≈117.0	≈10.2	≈72.4	≈10.2
Polyvinylpyrrolidone	≈51.5	≈3.8	44.2	≈3.8	≈27.3	≈3.8
Sodium lauryl sulphate	U	U	U	U	U	U
Sodium stearyl fumarate	≈26.8	≈2.0	≈23.0	≈2.0	≈14.2	≈2.0
Film coating	≈53.6	≈4.0	≈46.0	≈4.0	≈28.4	≈4.0
∑	1340.0	100.0	1150.0	100.0	710.0	100.0

U—Unknown.

**Table 2:** Preparation of calibration solutions.

Calibration Level	Concentration of the Stock Solution Used	Volume of Stock Solution Added	Volume of Water Added	Concentration of the Calibration Solution
	µg/mL	µL	µL	µg/mL
1	50.00 (stock solution 2)	80	9920	0.40
2		100	9900	0.50
3		200	9800	1.00
4		400	9600	2.00
5	500.0 (stock solution 1)	80	9920	4.00
6		120	9880	6.00
7		160	9840	8.00
8		200	9800	10.0

## Data Availability

The data presented in this study are available on request from the corresponding author.

## Supplementary Materials

The following supporting information can be downloaded at: https://www.mdpi.com/article/10.3390/pharmaceutics16081100/s1, Table S1. Sodium lauryl sulfate (CAS Number 151213; UNII 368GB5141J) in dosage forms based on the Inactive Ingredient Search for Approved Drug Products (FDA) [2].
