# Supplementary material for: A Method for the Colorimetric Quantification of Sodium Lauryl Sulphate in Tablets: A Proof of Concept
Source: Pharmaceutics. 2024 Aug 21;16(8):1100. doi: 10.3390/pharmaceutics16081100 (PMC11360801; doi:10.3390/pharmaceutics16081100)
Supplement: Supplementary file 1 [file pharmaceutics-16-01100-s001.zip › pharmaceutics-3136635-supplementary.pdf]

Table S1. Sodium lauryl sulfate (CAS Number 151213; UNII 368GB5141J) in dosage forms based on the Inactive Ingredient Search for Approved Drug Products (FDA) [2].

Route	Dosage Form	Max Potency per unit dose	Maximum Daily Exposure (MDE)
BUCCAL	TABLET	5.18mg	
DENTAL	GEL	1.47%w/w	
DENTAL	PASTE	1.5%w/w	
DENTAL	PASTE, DENTIFRICE	1.4%w/w	
ORAL	CAPSULE		600mg
ORAL	CAPSULE, COATED PELLETS	0.06mg	
ORAL	CAPSULE, COATED, EXTENDED RELEASE	NA	
ORAL	CAPSULE, DELAYED RELEASE		46mg
ORAL	CAPSULE, DELAYED RELEASE PELLETS		99mg
ORAL	CAPSULE, EXTENDED RELEASE		166mg
ORAL	DROPS	NA	
ORAL	GRANULE		12mg
ORAL	PELLET		2mg
ORAL	POWDER, FOR SUSPENSION		64mg
ORAL	SUSPENSION	705mg	
ORAL	SUSPENSION, EXTENDED RELEASE	0.08mg/1ml	
ORAL	TABLET		233mg
ORAL	TABLET, CHEWABLE		6mg
ORAL	TABLET, CHEWABLE, EXTENDED RELEASE		1mg
ORAL	TABLET, COATED		123mg
ORAL	TABLET, DELAYED RELEASE		199mg
ORAL	TABLET, EFFERVESCENT	1.5mg	
ORAL	TABLET, EXTENDED RELEASE		320mg
ORAL	TABLET, FILM COATED		240mg
ORAL	TABLET, FILM COATED, EXTENDED RELEASE	10.5mg	
ORAL	TABLET, FOR SUSPENSION		264mg
ORAL	TABLET, ORALLY DISINTEGRATING		16mg
ORAL	TABLET, ORALLY DISINTEGRATING, DELAYED RELEASE		3mg
RESPIRATORY	CAPSULE	NA	
RESPIRATORY	POWDER	NA	
SUBLINGUAL	TABLET	1.1mg	
TOPICAL	CREAM		214mg
TOPICAL	GEL	0.05%w/w	
TOPICAL	LOTION	0.5%w/w	
TOPICAL	OINTMENT		14mg
TOPICAL	SHAMPOO	65%w/w	
TOPICAL	SHAMPOO, SUSPENSION	40%w/v	

Route	Dosage Form	Max Potency per unit dose	Maximum Daily Exposure (MDE)
TOPICAL	SPONGE	5%w/w	
TOPICAL	SPRAY		7mg
VAGINAL	CREAM		12mg
VAGINAL	GEL	0.2%w/w	
VAGINAL	INSERT		15mg
VAGINAL	POWDER	3mg	
